# Endoscopic removal of fractured pancreatic duct stent from side-hole using drill dilator: A troubleshooting case

**DOI:** 10.1055/a-2744-8826

**Published:** 2025-12-17

**Authors:** Tomoya Takahashi, Yusuke Takasaki, Anna Fujiyama, Haruka Hagiwara, Yasuhisa Jimbo, Toshio Fujisawa, Hiroyuki Isayama

**Affiliations:** 1 73362 Department of Gastroenterology, Graduate School of Medicine, University of Juntendo, Tokyo, Japan; 2 12737 Department of Gastroenterology, Graduate School of Medicine, Chiba University, Chiba, Japan; 3 Division of Gastroenterology, Department of Medicine, Faculty of Medicine, Chulalongkorn University, Bangkok, Thailand

Endoscopic management of fractured pancreatic duct stents (PDSs) remains challenging, although several techniques have been reported 1 2 3 4 . We describe a case in which a fractured PDS was removed by engaging its side hole with a 0.018-inch guidewire (GW) and a drill dilator (TORNUS ES, Asahi Intec, Japan).

A 47-year-old man with chronic pancreatitis had two 7-Fr PDSs placed. The first PDS was removed with forceps, but the second PDS could not be removed and fractured when its distal flap became trapped at a stone/stricture. The fractured stent protruded slightly from the ampulla with visible side holes. Attempts at removal with forceps were abandoned due to the risk of further fracture. We therefore decided to pass the GW into the stent through a side hole. A 0.025-inch GW (Endoselector, Boston Scientific Japan, Japan) could not be passed, but a 0.018-inch GW (Fielder, Olympus, Japan) was successfully inserted through a side hole ( Fig. 1 , Fig. 2 ). Over this wire, the drill dilator was advanced into the stent with controlled torque, firmly engaging the lumen. The stent was then withdrawn into the scope and removed safely. The stent withdrawn into the scope and removed safely ( Video 1 ).

**Fig. 1:**
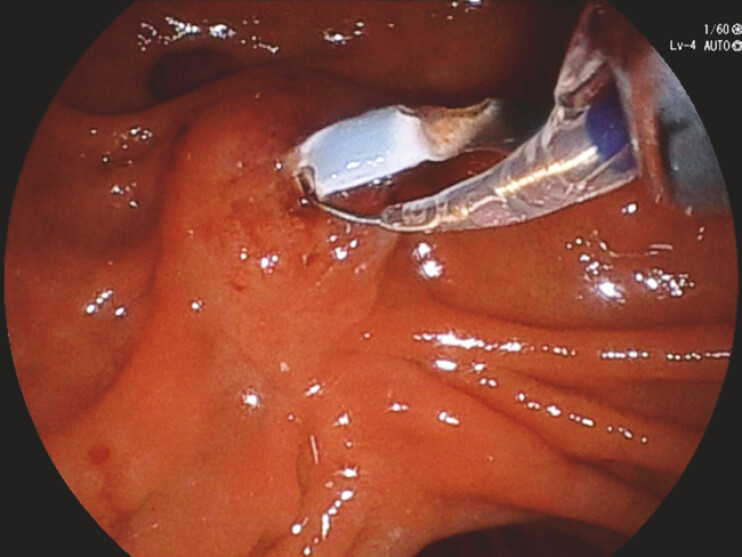
A 0.018-inch guidewire was inserted into the side hole using a tapered balloon catheter.

**Fig. 2:**
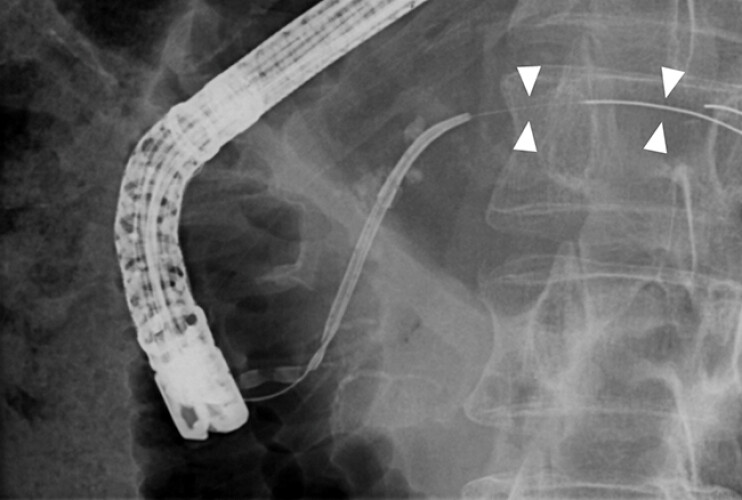
By exchanging a 0.018-inch guidewire, we successfully inserted the wire through the side hole.

Endoscopic removal of the fractured pancreatic duct stent from the side hole using the drill dilator.Video 1

Reports of the PDS removal using Tornus are limited 5 . To our knowledge, this is the first case in which a fractured PDS was removed by inserting Tornus through a side hole. The key was the successful GW insertion into the stent cavity via the side hole. A Soehendra dilator (Cook, USA), another type of drill dilator, was unsuitable because of its larger diameter. The Tornus provided strong anchoring within the lumen, suggesting that even stacked stents may be removable. Another benefit was that the removal power was strong with a drill dilator because it firmly embedded into the stent lumen, and a stacked stent may be removed successfully.

This case highlights a novel troubleshooting strategy for fractured PDS removal when conventional methods fail.

Endoscopy_UCTN_Code_CPL_1AK_2AC

## Notes

Endoscopy E-Videos https://eref.thieme.de/e-videos
*E-Videos* is an open access online section of the journal *Endoscopy* , reporting on interesting cases and new techniques in gastroenterological endoscopy. All papers include a high-quality video and are published with a Creative Commons CC-BY license. Endoscopy E-Videos qualify for HINARI discounts and waivers and eligibility is automatically checked during the submission process. We grant 100% waivers to articles whose corresponding authors are based in Group A countries and 50% waivers to those who are based in Group B countries as classified by Research4Life (see: https://www.research4life.org/access/eligibility/ ). This section has its own submission website at https://mc.manuscriptcentral.com/e-videos .

## References

[1] Kawaguchi Y Lin JC Kawashima Y Risk factors for migration, fracture, and dislocation of pancreatic stents Gastroenterol Res Pract 2015 2015 365457 10.1155/2015/365457 25945085 PMC4402177

[2] Ishigaki K Hamada T Isayama H Endoscopic removal of a proximally migrated pancreatic stent using a gooseneck snare Endoscopy 2014 46 E283 E284 10.1055/s-0034-1365792 24906102

[3] Ushio M Tomishima K Ishii S Successful withdrawal of migrated pancreatic stent with a prototype guiding sheath Endoscopy 2023 55 E5 E6 36084939 10.1055/a-1907-4640 PMC9812664

[4] Takahara N Isayama H Sasahira N Endoscopic removal of a piece of retained pancreatic stent with a novel new technique: turned guide-wire looping method Endoscopy 2012 44 E401 23169035 10.1055/s-0032-1309897

[5] Yanaidani T Matsumori T Muramoto Y Pancreatic stent removal with a novel drill dilator VideoGIE 2024 9 243 246 38766401 10.1016/j.vgie.2024.01.007 PMC11099314

